# Reduced temporal organization of narrative recall in adults with moderate-severe traumatic brain injury

**DOI:** 10.1016/j.cortex.2025.06.007

**Published:** 2025-07-01

**Authors:** Sharice Clough, Melissa J. Evans, Melissa C. Duff, Sarah Brown-Schmidt

**Affiliations:** a Multimodal Language Department, Max Planck Institute for Psycholinguistics, the Netherlands; b Department of Hearing and Speech Sciences, Vanderbilt University Medical Center, United States; c Department of Communication Sciences and Disorders, University of Iowa, United States; d Department of Psychology and Human Development, Vanderbilt University, United States

**Keywords:** Traumatic brain injury, Narrative discourse, Temporal contiguity, Organization, Declarative memory

## Abstract

Narrative discourse impairments are well documented in individuals with moderate-severe traumatic brain injury (TBI). Studies of narrative discourse (i.e., story generation, story retelling) in this population have frequently focused on impairment of semantic relations across utterances and the larger discourse context (e.g., cohesion, coherence, story grammar). Less attention has been given to the temporal organization of narrative retelling in TBI. We applied temporal contiguity analyses, a technique traditionally used to characterize temporal organization of free recall of wordlists, to quantify the temporal organization of participants’ story retellings with respect to the order in which the narrator originally presented the story details. We also conducted a parallel analysis of temporal contiguity of wordlist recall using data from the Rey Auditory Verbal Learning test. Participants with moderate-severe TBI and non-injured peers demonstrated above chance temporal organization and a tendency to make short transitions in the forward direction when recalling items in both the narrative recall and wordlist recall task. However, these effects were significantly reduced in the TBI group. Overall, their free recall performance was less temporally clustered, and they were more likely to make larger jumps between story details (or words in the wordlist recall task) than their non-injured peers when recalling stories. Examining free recall at multiple timepoints revealed that while repetition (i.e., multiple presentations of the wordlist) increased temporal organization of recall, long delays (i.e., one week) decreased temporal organization for both the TBI and non-injured groups. We propose that reduced temporal organization of narrative recall in individuals with moderate-severe TBI is linked to impairments in the declarative relational memory system. In line with retrieved-context models of free recall, memory disruption not only impacts the total number of story details recalled, but also the ability to use temporal context to encode and retrieve items in a sequentially organized way.

## 1. Introduction

Much of our daily life consists of stories. We might recount a funny episode from work to a friend or retell a story we originally heard from another source. The ability to organize narratives during story retelling is a critical skill for social communication. Stories have an underlying temporal structure with a beginning, middle, and end, consisting of a sequence of events. Individuals with traumatic brain injury (TBI) demonstrate difficultly with several aspects of narrative discourse production, including cohesion, coherence, and inclusion of essential details (Togher et al., 2014). These measures largely characterize how *semantically organized* or complete a speaker’s narrative is but focus less on the *temporal organization* of their retellings. The current study presents a novel application of a technique for characterizing the temporal organization of narrative recall in adults with moderate-severe TBI. Temporal contiguity (or the presence of strong temporal organization) reflects the tendency of participants to recall items together that were presented close in time at encoding. Items encoded together become associated due to their shared temporal context so that one item facilitates the retrieval of the other (Howard & Kahana, 1999, 2002; Kahana, 1996). We leverage temporal contiguity analyses (traditionally used in serial wordlist recall tasks) to quantify the temporal organization of participants’ narrative retellings. We examined whether participants with and without TBI recalled story details in an order that reflected the original order in which the narrator presented the story details. We discuss how underlying memory disruptions contribute to impaired temporal organization of narratives.

### 1.1. Measuring narrative organization

Individuals with TBI often have communication difficulties that are evident at the discourse level. In *narrative discourse* tasks, participants are asked to generate or retell a story. These narrations are often analyzed in terms of semantic organization. Researchers frequently characterize the extent to which speakers a) use relational elements like pronouns, conjunctions, and lexical items to bridge meaning across utterances (i.e., cohesion), b) maintain continuity of meaning across the discourse (i.e., coherence), and c) logically arrange episodes in a narrative in relation to cause-effect relationships (i.e., story grammar). It is well accepted that individuals with TBI can present with a variety of narrative discourse impairments that disrupt their ability to convey story details in a complete, efficient, clear, and semantically organized manner (for reviews, see Coelho et al., 2005; Lê & Coelho, 2023; Togher et al., 2014). Such studies tend to quantify the amount (e.g., frequency, ratio, or percentage) of cohesion or coherence errors across utterances (Carlomagno et al., 2011; Coelho et al., 2012, 2013; Davis & Coelho, 2004; Galetto et al., 2013; Glosser & Deser, 1991; Hartley & Jensen, 1991; Marini et al., 2011, 2014, 2017; Mentis & Prutting, 1987; Peach & Coelho, 2016; Peach & Hanna, 2021). Others use subjective rating scales to characterize how related a given utterance is to the previous utterance or the overall narrative theme (Coelho et al., 2012, 2013; Glosser & Deser, 1991; Hough & Barrow, 2003; Van Leer & Turkstra, 1999; Wright et al., 2013). Measures of story grammar tend to quantify the proportion of utterances organized into complete episodes (i.e., sequences of events containing an initating event, attempt by character to achieve a goal, and direct consequence of attempt) (Coelho et al., 2012; Coelho, 2002; Hay & Moran, 2005; Peach & Hanna, 2021).

These discourse measures may indirectly reflect some aspects of temporal organization (e.g., whether two sequential utterances are semantically related or whether story details are sequenced in a logical order). However, current approaches to narrative discourse analysis lack an ability to objectively quantify temporal organization. In contrast, temporal organization of free recall has long been of interest in the field of cognitive neuroscience of memory (Murdock, 1962; Tulving, 1962). Measures of temporal organization have traditionally used wordlist recall paradigms where participants are sequentially presented with a list of unrelated words and are immediately prompted to recall the wordlist. Researchers then compare the order participants recalled the words to the order in which the words were originally presented. Temporal organization is a ubiquitous property of memory retrieval. The *temporal contiguity effect* reflects the strong tendency to recall sequences of words in an order based on their neighboring positions in the original wordlist (Howard & Kahana, 1999, 2002; Kahana, 1996). Recall order also demonstrates a reliable forward asymmetric pattern. Participants are more likely to recall items from the original wordlist in a forward compared to backward direction (e.g., after recalling the fourth word they heard, participants are more likely to next recall the fifth word than the third word, moving forward in wordlist order).

Temporal contiguity analyses offer a way to quantify temporal organization through lag-conditional response probabilities (lag-CRP) (Kahana, 1996) and temporal organization scores (Polyn et al., 2009), described in detail below (section 2.3). An increasing number of studies have applied these approaches to more ecologically valid memory stimuli including free recall of personal autobiographical events (Moreton & Ward, 2010), elements from a guided walking tour (Diamond & Levine, 2020), news stories (Uitvlugt & Healey, 2019), and conversation (Evans, 2025). Narrative retellings offer another opportunity to study differences in temporal order between recalled items (i.e., the order in which an individual recalls story details) and studied items (i.e., the order in which a narrator originally presented the story details), thus providing a way to objectively quantify the temporal organization of recall using a semantically rich stimulus that more accurately reflects the memory demands of daily life.

Recently, our group applied these techniques to measure temporal organization of narrative recall in a rare group of individuals with amnesia who had bilateral hippocampal damage and selective severe declarative memory impairment (Evans et al., 2024). Individuals with amnesia, non-injured comparison participants, and a brain-damage comparison group with bilateral damage to the ventromedial prefrontal cortex (vmPFC) and no memory impairment retold four short stories immediately after hearing them. We found that individuals with amnesia recalled story details in a temporal order that reflected the order of presentation at above chance levels. However, their temporal contiguity was significantly attenuated relative to non-injured and brain-damage comparison groups. In other words, individuals with amnesia were more likely to make larger jumps between successively recalled details. The observed reduced temporal organization of narrative recall in amnesia provides evidence that the hippocampal declarative memory system supports temporal organization of free recall. Given that declarative memory impairment is one of the most common consequences of TBI (Monti et al., 2013; Morrow et al., 2020; Palacios et al., 2013; Rabinowitz & Levin, 2014; Rigon et al., 2020; Vakil, 2005), it is likely that individuals with TBI also have difficulty with temporal organization of narratives. However, temporal organization of narrative recall in TBI has not yet been objectively measured. In addition, whereas the individuals with amnesia have focal lesions to the hippocampi and selective memory impairment, individuals with TBI present with heterogenous patterns of diffuse neural injury and cognitive impairments (Covington & Duff, 2021; Hayes et al., 2016). Thus, disruptions to multiple cognitive processes likely contribute to impaired narrative organization in this group. We review these processes in the following section.

### 1.2. Cognitive correlates of impaired narrative organization

The diffuse nature of neuronal injury after TBI poses challenges for identifying the cognitive domains most strongly associated with impaired narrative organization. Executive function and working memory in particular have received much attention in describing discourse impairments in TBI (Brookshire et al., 2000; Chapman et al., 2006; Coelho et al., 2013; Coelho, 2002; Coelho et al., 1995; Galetto et al., 2013; Lê et al., 2012; Marini et al., 2014, 2017; Matsuoka et al., 2012; Mozeiko et al., 2011; Peach & Hanna, 2021; Youse & Coelho, 2005). Executive function comprises a set of higher-order cognitive capacities involving planning, organizing, inhibiting, and monitoring behavior for goal-oriented tasks (Lezak, 1995). Working memory is a temporary store where information can be processed and manipulated and is often considered a subcomponent process of executive function (Baddeley, 1992). These abilities are classically linked to damage to the frontal lobe and prefrontal brain regions (Alvarez & Emory, 2006; Stuss & Alexander, 2000) which are particularly susceptible to damage during closed head injury (Levin & Kraus, 1994).

Narrative discourse performance has been linked to cognitive ability more generally, drawing on multiple cognitive resources including executive function, working memory, and declarative memory (Coelho et al., 2013; Lê et al., 2012). However, these functions may contribute to different aspects of narrative organization. Declarative memory may play a larger role in narrative discourse when the task requires recalling a previously heard story (Togher et al., 2014) and involves representations of characters and events across space and time (Lê et al., 2012). On the other hand, executive function may play a larger role in generating the story from a stimulus such as a picture (Brookshire et al., 2000; Youse & Coelho, 2005). Lê et al. (2012) reference the structure building framework (Gernsbacher, 1990, 1996) to explain the relationship between multiple cognitive processes and narrative discourse, where declarative memory lays a foundation for mapping incoming information onto existing mental representations of the story, working memory temporally stores story information to facilitate mapping, and executive function shifts mental representations to accommodate incoming information or inhibit irrelevant information (Coelho et al., 2013; Lê et al., 2012; Mozeiko et al., 2011). This framework has been used to explain disordered discourse in other clinical populations with impairments in general cognitive processes, like schizophrenia (Gernsbacher et al., 1999).

Compared to executive function and working memory, the role of the declarative memory system in narrative discourse organization has received less attention in TBI (Lê et al., 2012). However, there is growing interest in links between the hippocampal declarative memory system and narrative organization in the field of cognitive neuroscience. Hippocampal activity is thought to be reinstated during recall to integrate related events and support coherent narrative structure (Baldassano et al., 2017; Cohn-Sheehy, 2023; Cohn-Sheehy et al., 2021). The hippocampal declarative memory system supports our ability to encode and retrieve new autobiographical memories for events and episodes of daily life, as well as semantic facts and general world knowledge (Cohen & Squire, 1980; Gabrieli et al., 1988). A central function of the hippocampus during new learning is relational binding which supports the encoding of co-occurring people, places, and things along with their spatial, temporal, and interactional relations (Cohen & Eichenbaum, 1993; Davachi, 2006; Eichenbaum & Cohen, 2001). Thus, the declarative, or relational, memory system (Cohen, 2015; Eichenbaum & Cohen, 2001; Rubin & Cohen, 2017) may play an important role not only in the ability to recall a complete set of details, but also in the ability to encode and retrieve relations amongst details that support the semantic and temporal organization of narratives.

Evidence from narrative discourse studies in individuals with severe and selective declarative memory impairment supports this claim. Our own group has shown that individuals with amnesia produced fewer cohesive ties across utterances and had lower ratings of local coherence in their narrative discourse samples (Kurczek & Duff, 2011). Further, they exhibit reduced temporal organization of narratives relative to comparison participants, as described above (Evans et al., 2024). Impaired discourse coherence has also been observed in other populations with memory impairment including dementia and amnestic mild cognitive impairment (Dijkstra et al., 2004; Kong et al., 2023; Seixas-Lima et al., 2020). In contrast, participants with focal damage to the vmPFC, an area of the prefrontal cortex associated with executive functions related to social judgements, emotional regulation, and decision making (Gläscher et al., 2012), did not show impairments in narrative discourse cohesion and coherence (Kurczek & Duff, 2012) or temporal organization (Evans et al., 2024). Kurczek and Duff (2012) proposed that some of the observed narrative discourse deficits in previous studies in populations with diffuse neural damage where memory impairment is a hallmark (e.g., TBI, AD), may have been misattributed to frontal lobe dysfunction and are instead attributable to the deficits in the hippocampal declarative memory system. Hippocampal pathology is common after TBI due to common occurrence of sequelae like hypoxia, anoxia, and seizure activity (Palacios et al., 2013; Tate & Bigler, 2000). In addition, pervasive diffuse axonal injury in TBI results in overall reduced connectivity of white matter tracts that disrupts processing and integration of information across major brain networks (Hayes et al., 2016; Sharp et al., 2014). These unique mechanisms of injury put individuals with TBI at high risk for declarative memory impairment.

Reduced temporal contiguity of wordlist recall has been documented in schizophrenia (Polyn et al., 2015) and amnesia (Palombo et al., 2019), phenomena which share a common hippocampal pathology. Applying these measures to narrative recall offers a new direction for examining the relationship between declarative memory and recall organization in populations with memory impairment. Retrieved-context models propose that presented items (i.e., words in a list or details in a story) are associated with a gradually changing internal contextual representation during encoding which subsequently guides memory search during free recall (Howard & Kahana, 2002; Kahana et al., 2008; Lohnas et al., 2015; Polyn et al., 2009). Encoding and retrieval of this temporal context is posited to be a fundamental function of the declarative memory system that is critical for temporal organization of recall. The current study draws on robust evidence from theoretical and computational models of human memory and observed reduced temporal organization of narrative recall in amnesia (Evans et al., 2024). We hypothesize that populations with memory impairment, including TBI, exhibit impaired narrative discourse organization due to any underlying disruption in the ability to encode and/or retrieve temporal context to guide their recall of story details in a way that reflects their original order.

### 1.3. The current study

The current study is a re-analysis of data described in our earlier work (Clough et al., 2023) in which the original aim was to examine how participants with TBI integrate information from a narrator’s gesture into their memory for stories. We found that participants with TBI did not significantly differ from non-injured participants in their likelihood of reporting unique information from the narrator’s gesture in their narrative retellings (e.g., retelling, “He looked for a new recipe *online*” after seeing the narrator produce a typing gesture paired with, “He searched for a new recipe”). The current study makes use of this dataset to examine the temporal organization of narrative recall in individuals with moderate-severe TBI and non-injured peers.

We compare the temporal organization of free recall in participants with and without TBI on two tasks: 1) narrative recall of short stories from the study described above, and 2) wordlist recall from the Rey Auditory Verbal Learning (AVLT) test administered to a large subset of the same participants in a separate session. To our knowledge, this is the first study to conduct a temporal contiguity analysis of both wordlist and narrative recall in an overlapping sample of participants. This sheds new insight into between-group differences in temporal organization using both traditional wordlist paradigms and more naturalistic discourse contexts. Further, both the wordlist and narrative recall tasks have multiple recall timepoints associated with them. This allows us to examine changes to temporal organization of free recall as a function of the passing of time and stimulus repetition.

The current study addresses the following research questions:

- Do adults with moderate-severe TBI recall items in an order that reflects the temporal organization of the original presentation order?
- Are findings of reduced or intact temporal organization in TBI consistent across wordlist and narrative recall tasks?
- Does temporal organization decrease over time for participants with and without TBI?
- Does temporal organization increase with repeated presentations of the stimulus items for participants with and without TBI?

To answer these questions, we compare groups with and without TBI on two measures of temporal organization: 1) *Temporal organization scores* which provide a single value that summarizes how temporally clustered an individual’s recall attempt is compared to the original presentation order, and 2) *lag-based conditional response probabilities* (Lag-CRP) which quantify the size and direction of transitions participants make between successively recalled items. Similar to individuals with hippocampal amnesia (Evans et al., 2024), we predicted that individuals with TBI would show above chance temporal organization scores. However, we predicted that their temporal organization scores would be significantly lower than non-injured peers and that they would show a decreased tendency to make short transitions between successively recalled items (i.e., making larger jumps between items). We expected this pattern to be similar for both the narrative recall and wordlist recall tasks (https://osf.io/rh2bs). Although not preregistered, we predicted that delays would have a negative impact on temporal organization in line with findings from previous temporal contiguity analyses (Healey et al., 2019). This is especially relevant for individuals with traumatic brain injury whose recall may be disproportionately negatively affected by delays relative to non-injured peers (Morrow et al., 2023). We predicted that repeated presentation and retrieval practice of the stimulus items would increase temporal organization, strengthening the associations between items and their temporal context in the original presentation order (Hong et al., 2024).

## 2. Methods

### 2.1. Participants

Participants were 60 adults with chronic (≥6 months post injury) moderate-severe TBI and 60 demographically matched non-injured comparison (NC) participants recruited from the Vanderbilt Brain Injury Patient Registry (Duff et al., 2022). Participants were matched pair-wise on sex, age, and education (Table 1). NC participants completed a medical history to rule out diagnoses and medications that can interfere with cognition (e.g., neurological or psychiatric conditions, developmental or learning disorders, untreated diabetes or sleep apnea). All participants with TBI sustained their injuries in adulthood and met the inclusion criteria for moderate-severe TBI using the Mayo Classification System (Malec et al., 2007). See Clough et al. (2023) for full description of participants’ injury etiologies and severity criteria.

### 2.2. Procedure

#### 2.2.1. Narrative recall task

**Fig. 1 –:**
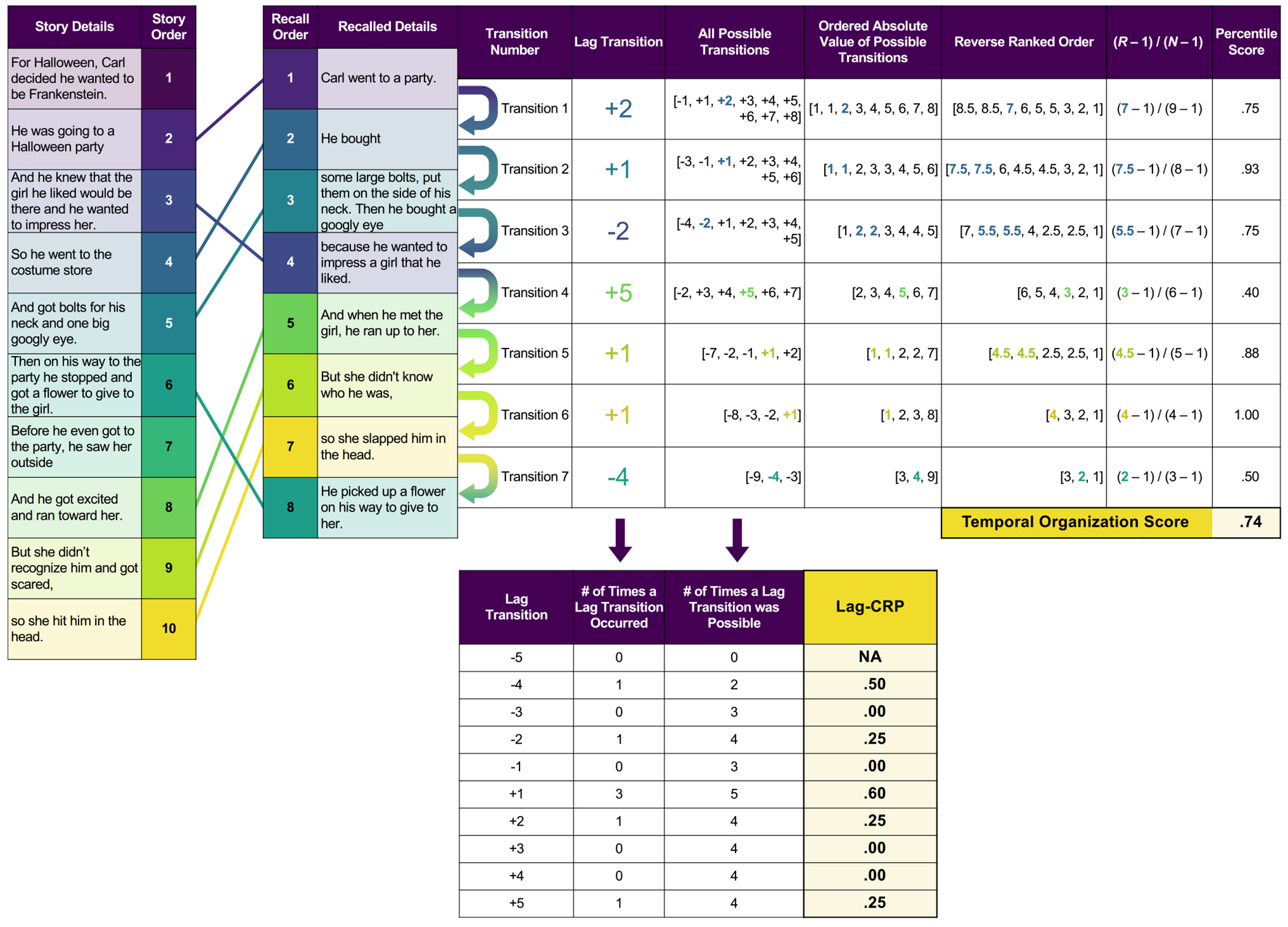
Example of temporal coding and calculations from a narrative retelling of a participant with TBI. The left table shows the order in which participants heard the story details (*Story Order*). These details are connected by colored lines corresponding to the order in which the participant recalled the details (*Recall Order*). Each pair of successively recalled details marks a transition (*Transition Number*). The value of the *Lag Transition* is the difference in the *Study Order* between two successively recalled details. For example, this participant first recalled Story Detail #2 followed by Story Detail #4, indicating a +2 Lag Transition. The list of *All Possible Transitions* is all possible lag transition values that could have occurred for a given transition based on the Story Details that had not yet been recalled at that point. The absolute values of these possible transitions are then ordered from smallest to largest, reflecting a distribution of possible temporal distances. These temporal distances are then assigned a reverse ranked order so that smaller temporal distances correspond to higher rank values. If there is a tie (e.g., two temporal distances of 1 are possible because both a +1 and −1 transition could have occurred), the mean rank is shared for the tied values. For each given transition, a *Percentile Score* is calculated by formula (*R* − 1)/(*N* − 1), where *R* is the rank value of the lag transition that did occur, and *N* is the number of all possible transitions. The mean of percentile scores for all transitions is the *Temporal Organization Score*. The bottom table indicates how *Lag-CRP* values are calculated. For lag transition values ranging from −5 to +5, the lag conditional response probability of each transition is calculated by dividing the number of times a lag transition occurred in the free recall series by the number of times that lag transition was possible throughout that entire recall series.

All participants provided informed consent prior to participating, in accordance with Vanderbilt University Medical Center IRB guidelines. Participants watched videos of a female adult native English speaker narrating four stories about a man named Carl who experiences a string of bad luck. Each story was about 30 sec long, consisted of six sentences, and contained a maximum of 10–12 story details (see Fig. 1 for an example). The details were determined a priori in Clough et al. for the purpose of calculating recall accuracy. Decisions about detail boundaries were guided by the presence of independent clauses and identification of idea units representing the gist of each relevant scene or plot description (see scoring guide on OSF project for full description of story details: https://osf.io/3fdsz/). During the stories, the narrator produced four gestures, two of which were redundant with information in speech (e.g., saying, “He formed the meat into balls” while producing a meatball-patting gesture) and two of which were complementary, adding new information not in the speech signal (e.g., saying, “He searched for a new recipe” while producing a typing gesture). This gesture manipulation was relevant for the original study goals in Clough et al. (2023) but did not factor into the current analysis. Participants retold each story three times: immediately after hearing the story, approximately 20 min later, and one week later. We refer to these as the No Delay, Short Delay, and Long Delay timepoints, respectively.

#### 2.2.2. Wordlist recall task

In a separate session, a subset of participants (57/60 participants with TBI and 50/60 NC participants) completed the Rey Auditory Verbal Learning Test (AVLT). During the AVLT, participants were auditorily presented with a list of 15 unrelated words at a rate of one word per second. Participants heard the wordlist five times in the same order and were asked to recall verbally as many words as possible after each list presentation (Trials 1 through 5). After a 20-min delay, participants were asked again to verbally recall as many words as possible from the list (Delayed Recall).

### 2.3. Recall coding

Clough et al. (2023) scored each narrative retelling for the number of story details participants recalled. Each story had a maximum of 10–12 details. For the current study, we added additional coding to indicate the order in which participants recalled the story details. Repetitions of story details were ignored.

Similarly, we coded the order in which participants recalled the 15 words from the AVLT. We coded recall order at Trial 1 (representing their immediate recall after hearing the wordlist for the first time), Trial 5 (representing their total recall after five sequential presentations of the wordlist), and the Delayed Recall timepoint (representing their recall maintenance after a short 20-min delay). Repetitions of words were ignored.

We compared the order in which the story details/words were presented with the order in which participants recalled the story details/words to quantify temporal organization across the two groups and three timepoints using two methods: percentile-rank temporal organization scores and lag-based conditional response probabilities.

#### 2.3.1. Percentile-rank temporal organization score

We first examined percentile-rank *temporal organization scores* (Polyn et al., 2009). Temporal organization scores quantify the magnitude of temporal clustering of a participants’ free recall with a single number, where a value of 1.0 reflects perfect temporal organization and .5 reflects chance-level organization (Sederberg, Miller, W. Howard, & Kahana M, 2010; Hong et al., 2024; Polyn et al., 2009). Fig. 1 shows an example of how temporal organization scores are calculated for a given narrative retelling. Each pair of successively recalled items by the participant marks a transition. For a given transition, the difference between the study order position of the two recalled details is called the *lag*. For example, if the participant first recalled the second story detail followed by the fourth study detail, they made a +2 lag transition. The sign (+/−) indicates whether the details were recalled in a forward or backward direction, respectively. For each transition, a percentile score is calculated that compares the temporal distance of two successively recalled items to that of a distribution of possible recalled items based on the set of items that had not yet been recalled (Polyn et al., 2009). A temporal organization score was computed for each participant, story, and timepoint as an average of percentile scores computed for each lag transition (see Fig. 1).

Temporal organization scores do not directly reflect how much of a story participants recalled. Rather, they reflect the degree of temporal clustering of their successively recalled details. Participants who make only +1 lag transitions between successively recalled details will have a perfect temporal organization score of 1.0, regardless of whether they recalled three or ten story details total. In contrast, two participants can recall the same number of story details (e.g., three), but have very different temporal organization scores. For example, Participant A might make short transitions between recalled items resulting in high temporal clustering [e.g., a recall order of (2, 1, 4) has a temporal organization score of .90] whereas Participant B might make larger jumps between recalled items resulting in low temporal clustering [e.g., a recall order of (1, 5, 9) has a temporal organization score of .38]. It should be noted that although Participant B exhibits low temporal clustering, they recall items in chronological order relative to the original presentation order. Thus, temporal organization scores can be negatively impacted by large jumps between successively recalled items even when items are recalled in order. To better illustrate the variability in temporal organization scores for different recall amounts, we provide example transcripts of pairs of narratives with high and low temporal organization scores across a range of different recall percentages (See Supplementary Materials A).

#### 2.3.2. Lag-based conditional response probability

Our second analysis examined temporal organization by calculating *lag-conditional response probabilities* (Lag-CRP). Lag values were converted into a *conditional response probability*, indicating the probability that participants made a given lag transition conditional on all possible lag transitions (Kahana, 1996). This calculation considers the overall study list length (i.e., number of story details or words in the list) and previous items that have already been recalled. For both narrative recall and wordlist recall, we truncated possible lag transitions to a range between − 5 and +5, as transitions beyond these are very infrequent. Because repetitions were ignored, a lag transition of 0 was not possible. Thus, for each participant, story, and timepoint, each lag transition (i.e., −5, −4, −3, −2, −1, +1, +2, +3, +4, +5) received a lag-CRP value that was calculated as the number of times a given lag transition occurred divided by the number of times it could have possibly occurred (See Fig. 1).

### 2.4. Analysis plan

We examined temporal organization of free recall across two different tasks: Narrative recall of story details in a reanalysis of the data from Clough et al. (2023) and wordlist recall from the AVLT. All analyses included a group (NC, TBI) factor and a timepoint factor (narrative task: No Delay, Short Delay, Long Delay; wordlist task: Trial 1, Trial 5, Delayed Recall). Significant effects of group indicate differences in the size or magnitude of effects between TBI and NC groups. Significant effects of timepoint indicate changes in the size or magnitude of effects over time (narrative task) and as a function of repeated practice (wordlist task). For all analyses, the NC group was dummy coded as the reference level. For the narrative recall task, the No Delay timepoint was dummy coded as the reference level. For the wordlist recall task, the Trial 5 timepoint was dummy coded as the reference level, where comparison of Trial 1 to Trial 5 indicates differences in temporal organization as function of repeated practice, and comparison of the Delayed Recall timepoint to Trial 5 indicates whether participants maintained temporal organization after a short delay. Main effects are interpreted as simple effects for the reference level conditions. To probe significant interactions, we reverse dummy coded the models when applicable to examine the direction and magnitude of effects at other reference levels. Analyses were conducted using the *lme4* (Version 1.1–35.2; Bates et al., 2015), *glmmTMB* (Version 1.1.9; Brooks et al., 2017), and *buildmer* (Version 2.11; Voeten, 2023) packages in R version 4.3.3 (R Core Team, 2024). Data and the analysis script are available on OSF (https://osf.io/3fdsz/).

#### 2.4.1. Recall accuracy

Do adults with TBI differ from non-injured peers in their total recall in the a) narrative and b) wordlist tasks? Does recall accuracy change over time within each task?

To compare total recall accuracy across groups, we used binomial mixed effect regression models with a logit-link function to predict the probability of recalling an item (i.e., story detail in the narrative recall task or word in the wordlist recall task) as a function of participant group, timepoint, and their interactions. The random effects structure was determined by *buildmer* which identifies the largest possible regression model that will converge and uses stepwise elimination to find the most parsimonious model based on information criteria.

#### 2.4.2. Temporal organization scores

Do participants demonstrate above chance temporal clustering across all groups and timepoints in the a) narrative and b) wordlist recall tasks?

To assess whether temporal clustering was greater than chance, we used one-sample *t* tests to compare temporal organization scores to a chance level of .5 by group and by timepoint for both the narrative recall and wordlist recall tasks.

Do adults with TBI differ from non-injured peers in the magnitude of their overall temporal organization scores in the a) narrative and b) wordlist recall tasks? Do overall temporal organization scores change over time within each task?

To compare differences in the magnitude of temporal organization, we used a linear mixed effects model to predict temporal organization score as a function of group, timepoint, and their interactions. The random effects structure was determined by *buildmer*.

Supplementary analysis: are overall temporal organization scores associated with recall performance in the a) narrative and b) wordlist recall tasks?

To better understand the relationship between temporal organization scores and recall performance in each task, we ran a follow up model with the same structure as the previous analysis, but adding Percent Recall for each story/wordlist (grand mean-centered) as a covariate. This allowed us to examine effects of group and timepoint on temporal organization scores after accounting for differences in the amount recalled (see Supplementary Materials B).

#### 2.4.3. Lag-conditional response probability

Do adults with TBI differ from non-injured peers in the size or direction of transitions they make between successively recalled items in the a) narrative and b) wordlist recall tasks? Does the probability of making a given transition change over time within each task?

To assess whether participants with TBI differed from noninjured participants in the size or direction of their recall transitions, we predicted the magnitude of CRP values as a function of group, timepoint, absolute lag transition (|1|, |2|, |3|, |4|, |5|), and transition direction (backward = −.5, forward = .5).

Because for a given recall attempt, many lag transitions were not made (e.g., |4| and |5| transitions were very unlikely), the data was heavily zero-inflated (8,637/12,473 observations were zero for the narrative recall task, and 683/1044 observations were zero for the wordlist recall task). Therefore, we chose to use a zero-inflated beta regression model from *glmmTMB* to account for these excess zeros. A beta distribution is appropriate for probability data that is restricted to values between 0 and 1 (i.e., 0 < *y* < 1). The zero-inflated beta regression additionally allows for values equal to zero (i.e., 0 ≤ *y* < 1). The zero-inflated beta regression models two processes: 1) The first distinguishes between zero and non-zero values (i.e., a logit-link function that predicts whether or not a given lag transition was made). 2) For non-zero values, the second model uses the beta distribution to predict CRP probabilities (i.e., a log-link function that predicts the magnitude of CRP values provided that a given lag-transition was made). It should be noted that our dataset also contained some one values, indicating that the participant made a given lag transition every time it was possible in their recall attempt (e.g., they made all +1 transitions). Because values of exactly one are not permissible in beta regression or zero-inflated beta regression, one solution is to use a zero-one inflated beta regression, where it models a third process that distinguishes ones from non-ones. However, because CRP values of one were relatively rare (336/12,473 observations were one for the narrative recall task, and 12/1044 observations were one in the wordlist recall task), we chose to adjust these values to .999, rather than model this process separately. Because the zero-one inflated *glmmTMB* model was not compatible with the *buildmer* function, we used the *anova* function in R to compute a likelihood ratio test to compare models with nested random effects structures to determine if more complex models significantly improved fit over simpler models to identify the most parsimonious random effects structure. The use of zero-inflated beta regression deviates from the pre-registered analysis plan in which we specified that we would model CRP values as continuous only but was necessary to account for the notable frequency of 0s in the data.

## 3. Results

### 3.1. Recall accuracy

#### 3.1.1. Narrative recall task: do adults with TBI differ from non-injured peers in their total recall? Does recall accuracy change over time?

**Fig. 2 –:**
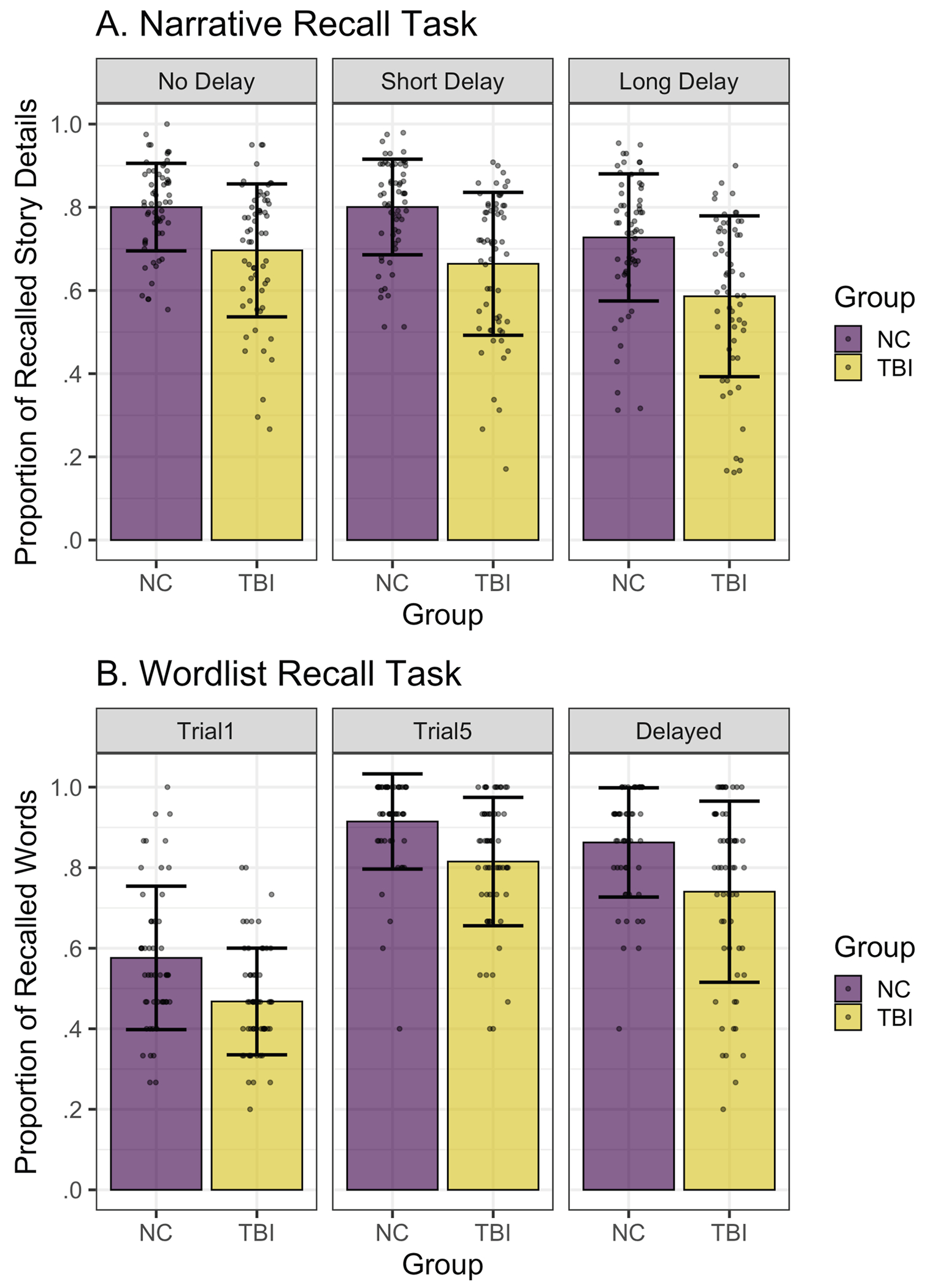
Proportion of recalled items across groups and timepoints in the A) narrative recall task and B) wordlist recall task (AVLT). Each dot represents the proportion of items a participant recalled (averaged across the four stories in the narrative recall task). Bars represent one standard deviation of the mean.

Fig. 2a depicts the mean proportion of recalled story details across groups and timepoints in the narrative recall task. We used a binomial mixed effects model to predict the probability of recalling a story detail as a function of group and timepoint with random intercepts for participant and story (Table A1). Participants with TBI were significantly less likely to recall a story detail than their non-injured peers at the No Delay timepoint ($\hat{\beta }=-.596,z=-3.89,p<.001$). There was no significant effect of the Short Delay relative to No Delay on narrative recall in the NC group. However, there was a significant effect of the Long Delay ($\hat{\beta }=-.433,z=-6.23,p<.001$), where NC participants were significantly less likely to recall a story detail one week later compared to immediately after hearing the story. Interactions between group and timepoint were not significant, suggesting that like NC participants, participants with TBI were similarly likely to recall a detail at the Short compared to No Delay timepoint but demonstrated reduced recall at the Long Delay timepoint.

#### 3.1.2. Wordlist recall task: do adults with TBI differ from non-injured peers in their total recall? Does recall accuracy change with repeated practice?

Fig. 2b depicts the proportion of words recalled across groups and timepoints in the wordlist recall task (AVLT). We used a binomial mixed effects model to predict the probability of recalling a word as a function of group and timepoint with random intercepts for participant and word (Table A2). Participants with TBI were significantly less likely to recall a word than their non-injured peers at the Trial 5 timepoint ($\hat{\beta }=-1.023,z=-4.30,p<.001$). A significant effect of the Trial 1 timepoint relative to the Trial 5 timepoint $(\hat{\beta }=-2.421$, z=−14.90,p<.001) indicated that NC participants were significantly less likely to recall a word after hearing the wordlist the first time compared to after the fifth presentation of the wordlist. There was a significant interaction between group and the Trial 1 timepoint ($\hat{\beta }=-.474,z=-2.34,p=.02$). Results of the reverse-dummy coded model with the TBI group as the reference level indicated that the effect of the Trial 1 timepoint was also significant for the TBI group but smaller in magnitude than the NC group ($\hat{\beta }=-1.947,z=-15.63,p<.001$). A significant effect of the Delayed Trial timepoint ($\hat{\beta }=-.595$, z=−3.38,p<.001) indicated that NC participants were significantly less likely to recall a word after a short delay compared to the Trial 5 timepoint. There was no significant interaction between Group and the Delayed Trial timepoint, indicating that the effect of the Delayed Trial did not significantly differ for the TBI group.

Thus, participants with TBI demonstrated memory impairment in both the narrative recall and wordlist recall tasks relative to non-injured peers. Repetition improved recall accuracy in the wordlist recall task, and delays had a negative impact on recall accuracy in both tasks for both participant groups.

### 3.2. Temporal organization scores

#### 3.2.1. Narrative recall task: do participants demonstrate above chance overall temporal organization scores across groups and timepoints?

**Fig. 3 –:**
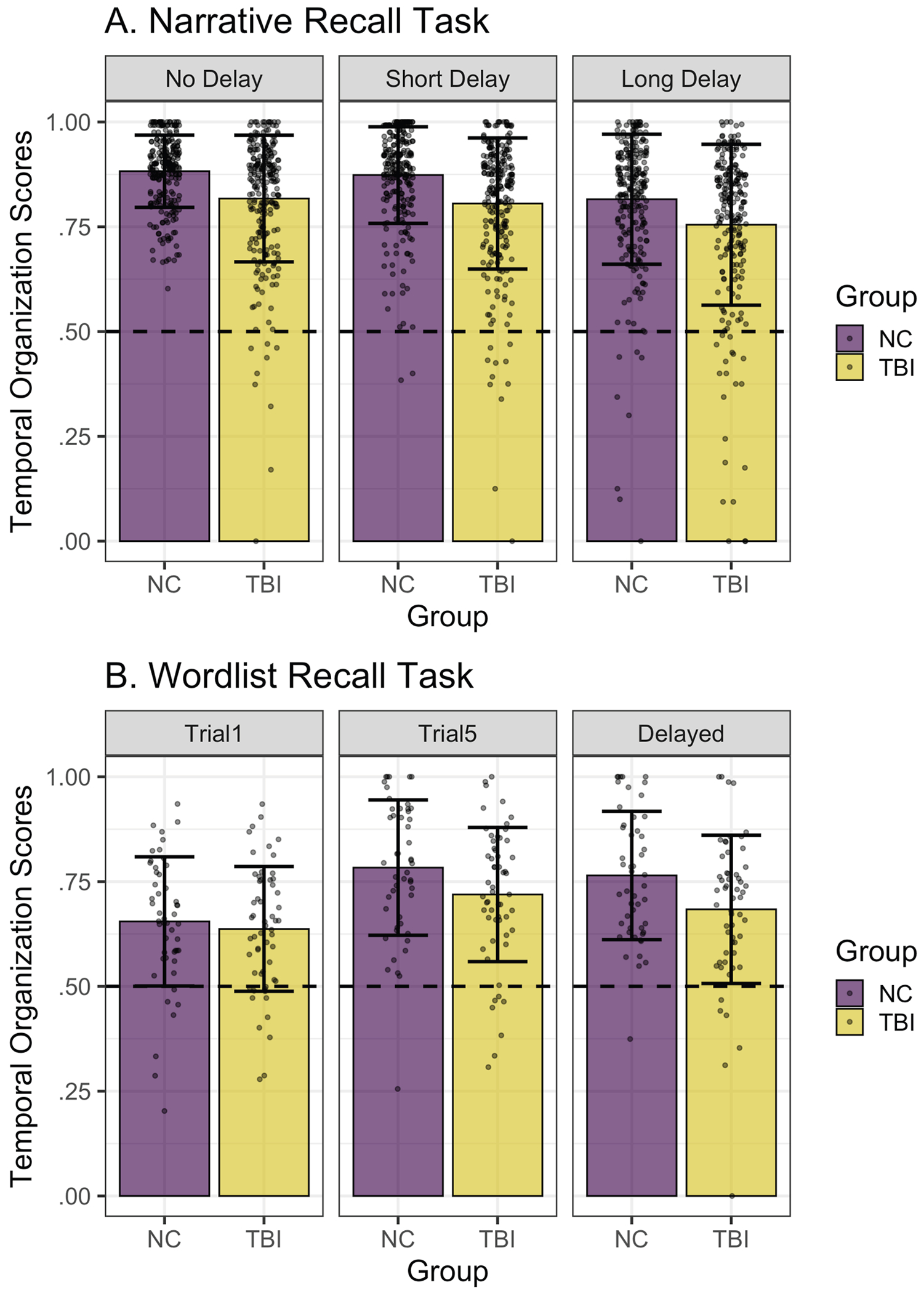
Mean temporal organization scores across groups and timepoints in the A) narrative recall task and B) wordlist recall task (AVLT). In the narrative recall task, each dot represents a participant’s retelling of one of the four stories. Bars represent one standard deviation of the mean. The dotted line indicates chance.

Fig. 3a depicts temporal organization scores across groups and timepoints in the narrative recall task. Both participants with and without TBI had temporal organization scores significantly above chance (Table 2). Temporal organization scores were also significantly above chance for each of the three timepoints.

#### 3.2.2. Narrative recall task: do adults with TBI differ from non-injured peers in the magnitude of their overall temporal organization scores. Do temporal organization scores change over time?

We used a linear mixed effects model to predict temporal organization scores as a function of group and timepoint with random intercepts for participant and story (Table A3). Participants with TBI had significantly lower temporal organization scores than NC participants ($\hat{\beta }=-.066,t=-3.60$, p<.001). NC participants showed no significant difference in the temporal organization scores between the No Delay and Short Delay timepoints; however, their narrative retellings had significantly lower temporal organization scores at the Long Delay (1 week later) compared to No Delay timepoint ($\hat{\beta }=-.067,t=-6.09,p<.001$). The lack of significant interactions between group and timepoints indicated that the effect of the Short and Long Delay on temporal organization scores followed a similar pattern in the TBI group.

In a follow up model, we added Percent Recall for each story as a covariate to explore the relationship between temporal organization scores and recall performance. When accounting for differences in narrative recall percentage, the effect of participant group was no longer significant; however, the effect of the Long Delay remained significant. This suggests that a one-week delay (but not the TBI group factor) had a negative impact on temporal organization above and beyond its impact on reduced recall performance (see Supplementary Materials B).

#### 3.2.3. Wordlist recall task: do participants demonstrate above chance overall temporal organization across groups and timepoints?

Fig. 3b depicts temporal organization scores across groups and timepoints in the wordlist recall task. Both participants with and without TBI had temporal organization scores significantly above chance (Table 3). Temporal organization scores were also significantly above chance for each of the three timepoints.

#### 3.2.4. Wordlist recall task: do adults with TBI differ from non-injured peers in the magnitude of their overall temporal organization scores? Do temporal organization scores change with repeated practice?

We attempted to fit a linear mixed effect model, but *Buildmer* determined all random effects should be removed. Thus, we used a linear regression model to predict temporal organization score as a function of group and timepoint with no random effects (Table A4). Participants with TBI had significantly lower temporal organization scores than NC participants at the Trial 5 timepoint ($\hat{\beta }=-.064,t=-2.08,p=.04$). NC participants’ wordlist temporal organization scores were significantly lower at the Trial 1 compared to Trial 5 timepoint ($\hat{\beta }=-.128,t=-4.02$, p<.001), indicating that NC participants showed significant improvements in temporal organization with multiple repetitions of the wordlist. A lack of significant difference between the Trial 5 and Delayed recall timepoint indicates that NC participants maintained temporal organization of wordlist recall after a short delay. The lack of significant interactions between participant group and timepoints indicated that the effect of repetition and short delay on temporal organization scores followed a similar pattern in the TBI group.

In a follow up model, we added Percent Recall for each wordlist as a covariate to explore the relationship between temporal organization scores and recall performance. When accounting for differences in wordlist recall percentage, the effect of participant group and Trial 1 timepoint were no longer significant, suggesting that these effects were not independent from memory performance in the task (see Supplementary Materials B).

### 3.3. Lag-conditional response probability

#### 3.3.1. Narrative recall task: do adults with TBI differ from non-injured peers in the size or direction of transitions they make between successively recalled items. Does the probability of making a given transition change over time?

**Fig. 4 –:**
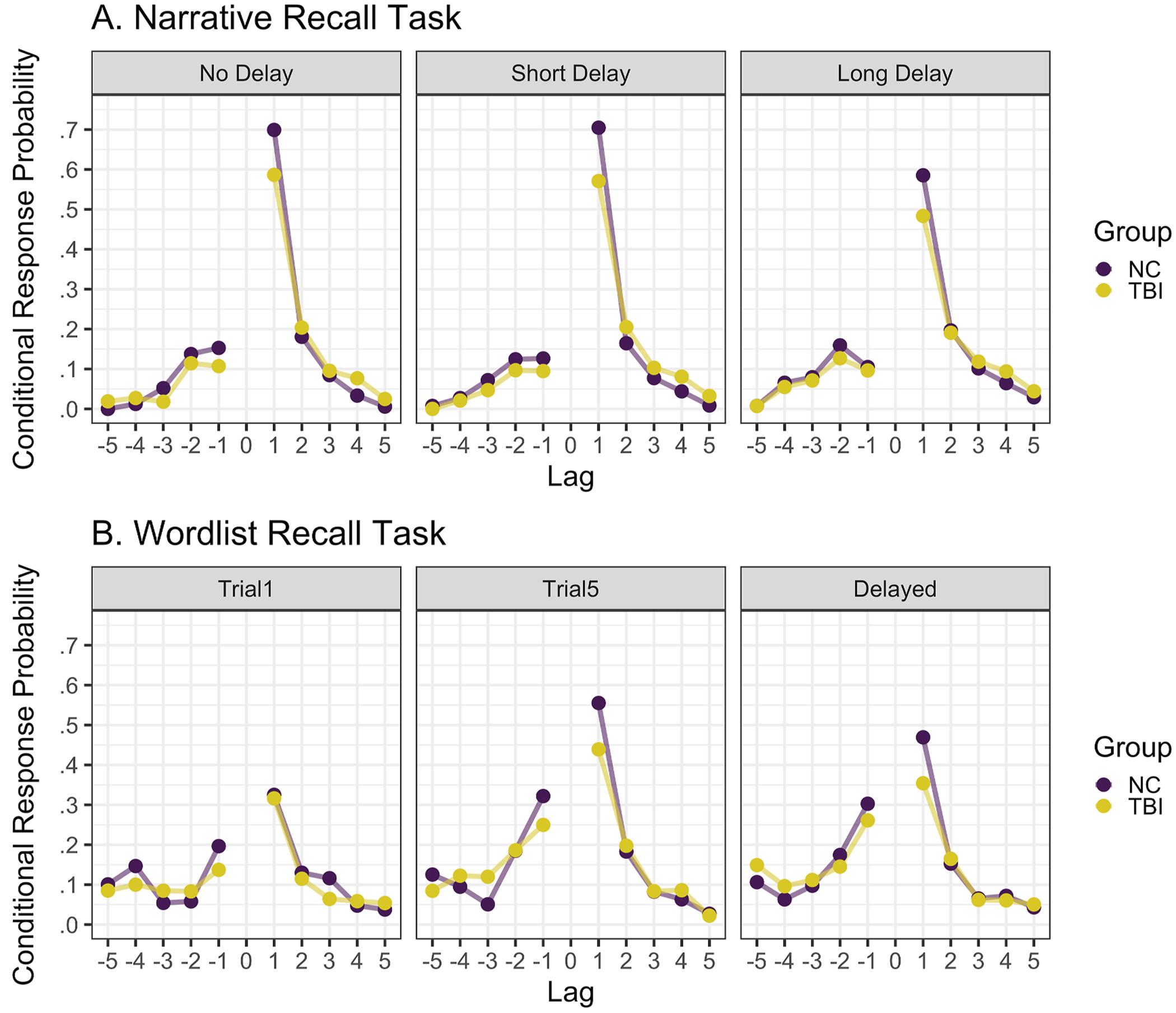
Mean conditional response probability across groups, timepoints, lag values, and directions in the A) narrative recall task and B) wordlist recall task (AVLT).

Fig. 4A depicts lag-conditional response probability values across groups and timepoints for the narrative recall task.

#### 3.3.2. Zero-inflation model

We modeled the probability that the CRP value for a given lag transition was zero (i.e., the lag transition was never made, despite being possible) or not (i.e., CRP >0) as a function of participant group, timepoint, lag, and direction with a random slope for lag by participant and a random slope for lag by story (Table A5).

There was a significant effect of |lag| on binary CRP ($\hat{\beta }=1.342,z=10.47,p<.001$), where longer lag transitions were significantly more likely to have a zero CRP value, indicating that NC participants more commonly made shorter transitions when recalling story details. There was also a significant effect of transition direction on binary CRP ($\hat{\beta }=-4.606,z=-13.42,p<.001$), where forward transitions were significantly less likely to have a zero CRP value than backwards transitions, indicating that NC participants tended to recall details in a forward direction. A significant |lag| by direction interaction ($\hat{\beta }=.804,z=5.70,p<.001$) indicated that the preference for shorter lag transitions over longer ones was more pronounced in the positive than the negative direction. These three effects within the NC group replicate the typical pattern of temporal contiguity found in the literature – that participants tend to make smaller forward lag transitions (Howard & Kahana, 2002; Kahana, 1996). There was no significant effect of the Short Delay on CRP values in the NC group; however, a significant effect of the Long Delay ($\hat{\beta }=.965$, z=4.38,p<.001) indicated that overall CRP values for a given lag transition were more likely to be zero one week later compared to immediately after hearing the story in the NC group. Similarly, there was no interaction between |lag| and the Short Delay timepoint in the NC group, but there was a significant interaction between |lag| and the Long Delay timepoint in the NC group ($\hat{\beta }=-.466,z=-5.38,p<.001$). To interpret this interaction, we reverse-dummy coded the model, setting the Long Delay timepoint as the reference level. There was also a significant effect of |lag| at the Long Delay timepoint ($\hat{\beta }=.875,z=7.379,p<.001$), but the magnitude was significantly reduced relative to the No Delay timepoint. Thus, the NC group was more likely to make longer rather than shorter lag transitions in their story retellings one week later compared to immediately after hearing the stories. Interactions between timepoint and transition direction as well as three-way interactions between timepoint, |lag|, and transition direction were not significant.

There was a significant effect of group ($\hat{\beta }=.856,z=3.10$, p=.002), indicating that participants with TBI were more likely to have a CRP value of 0 for a given lag transition than NC participants. There were no significant interactions between group and timepoint, indicating that the Short and Long Delay had a similar effect on the conditional response probabilities for both NC and TBI groups. A significant interaction between group and |lag| ($\hat{\beta }=-.320,z=-3.05,p=.002$) indicated that the effect of lag on CRP values significantly differed between the two groups. To interpret this interaction, we reverse-dummy coded the model, setting the TBI group as the reference level. Like the NC group, the TBI group demonstrated a significant effect of |lag| on binary CRP ($\hat{\beta }=1.022$, z=8.35,p<.001), but the magnitude of the effect was significantly smaller than their non-injured peers. Thus, the TBI group was more likely to make longer rather than shorter lag transitions relative to the NC group. There was no significant interaction between group and transition direction and all three- and four-way interactions with group, timepoint, |lag|, and transition direction were not significant.

#### 3.3.3. Beta regression model

The second part of the model predicts the CRP value for a given lag transition for all non-zero values (i.e., when a lag transition was made, how high was its probability?) as a function of participant group, timepoint, |lag|, and transition direction with random slope for |lag|, direction, and their interaction by participant and a random slope for |lag| by story (Table A6).

The effect of |lag| was not significant; whereas the length of a lag transition predicted whether a given transition occurred (see zero-inflation model above), it did not additionally predict the magnitude of the CRP value when a transition did occur. Like in the zero-inflation model, there was a significant effect of transition direction on CRP values ($\hat{\beta }=1.853,z=5.73$, p<.001), where transitions in the forward direction had higher probabilities than those in the backward direction in the NC group. Like in the zero-inflation model, a significant |lag| by direction interaction ($\hat{\beta }=-1.703,z=-8.90,p<.001$) indicated that a preference for shorter lag transitions over longer ones was more pronounced in the positive than the negative direction. There was no significant effect of the Short Delay on CRP magnitudes in the NC group, but there was a significant effect of the Long Delay ($\hat{\beta }=-.436,z=-2.38$, p=.02), indicating that NC participants tended to have lower overall conditional response probabilities one week later compared to immediately after hearing the story. There was no interaction between |lag| and timepoint in the NC group. The interaction between the Short Delay and transition direction was not significant; however, the interaction between the Long Delay and transition direction was significant ($\hat{\beta }=-.840,z=-2.29,p=.02$). To interpret this interaction, we reverse-dummy coded the model, setting the Long Delay timepoint as the reference level. Although the effect of transition direction was also significant at the Long Delay timepoint ($\hat{\beta }=1.013,z=3.33,p=.001$), the magnitude was significantly reduced relative to the No Delay timepoint, indicating that the tendency to recall details in a forward direction was less pronounced at the Long Delay compared to No Delay timepoint. Similarly, the three-way interaction between Short Delay, |lag|, and transition direction was not significant, but the three-way interaction between the Long Delay, |lag|, and transition direction was ($\hat{\beta }=.613,z=3.16$, p=.002). The results of the reverse-dummy coded model indicated that relative to the No Delay timepoint, the interaction effect between |lag| and transition direction was significant but smaller in magnitude ($\hat{\beta }=-1.090,z=-6.67$, p<.001), indicating a reduced tendency for smaller transitions in the forward direction after a one-week delay in the NC group.

There was a significant effect of group ($\hat{\beta }=-.609,z=-2.57$, p=.01), indicating that participants with TBI tended to have lower overall conditional response probabilities than NC participants. There were no significant interactions between group and timepoint, indicating that the Short and Long Delay had a similar effect on conditional response probabilities for both NC and TBI groups. There were also no significant interactions between group and |lag| or between group and direction. There was a significant three-way interaction between group, |lag| and direction ($\hat{\beta }=.559,z=2.17,p=.03$). The reverse-dummy coded model indicated that although the |lag| *direction interaction was also significant in the TBI group ($\hat{\beta }=-1.144,z=-6.53,p<.001$), indicating a preference for shorter lag transitions in the forward direction, the magnitude of the effect was significantly reduced relative to the NC group. All other three- and four-way interactions among group, timepoint, |lag|, and transition direction were not significant.

#### 3.3.4. Wordlist recall task: do adults with TBI differ from non-injured peers in the size or direction of transitions they make between successively recalled items? Does the probability of making a given transition change with repeated practice?

Fig. 4B depicts lag-conditional response probability values between groups and across timepoints for the wordlist recall task.

#### 3.3.5. Zero-inflation model

We modeled the probability that the CRP value for a given lag transition was zero (i.e., the lag transition was never made, despite being possible) or not (i.e., CRP >0) as a function of participant group, timepoint, |lag|, and direction with a random intercept for participant (Table A7).

There was a significant effect of |lag| on binary CRP ($\hat{\beta }=.789,z=9.03,p<.001$), where longer lag transitions were significantly more likely to have a zero CRP value, indicating that NC participants tended to make shorter transitions when recalling words. There was also a significant effect of transition direction on binary CRP ($\hat{\beta }=-1.839,z=-3.39,p=.001$), where forward transitions were significantly less likely to have a zero CRP value than backwards transitions, indicating that NC participants tended to recall words in a forward direction. A significant |lag| by direction interaction ($\hat{\beta }=.577$, z=3.31,p=.001) indicated that the preference for shorter lag transitions over longer ones was more pronounced in the positive than the negative direction. As with the zero-inflation model for the narrative recall task, this is typical of lag-CRP curve data. There was a significant effect of the Trial 1 timepoint on CRP values ($\hat{\beta }=1.124,z=3.11,p=.002$), where CRP values at the Trial 1 timepoint were significantly more likely to be zero than at the Trial 5 timepoint in the NC group; however, there was no significant effect of the Delayed Trial, indicating that CRP values were similarly likely to be zero between the Trial 5 timepoint and the Delayed Trial timepoint in the NC group. Similarly, there was a significant interaction between | lag| and the Trial 1 timepoint in the NC group ($\hat{\beta }=-.282$, z=−2.44,p=.02) but not between |lag| and the Delayed Trial timepoint. To interpret this interaction, we reverse-dummy coded the model, setting the Trial 1 timepoint as the reference level. Although the effect of |lag| was also significant at the Trial 1 timepoint ($\hat{\beta }=.507,z=6.66,p<.001$), the magnitude was significantly reduced relative to the Trial 5 timepoint. Thus, the NC group was more likely to make shorter lag transitions after five repetitions of the wordlist compared to hearing it for the first time. Interactions between the Trial 1 and Delayed timepoints and transition direction were not significant. The three-way interaction between Trial 1, |lag|, and transition direction was not significant; However, there was a significant three-way interaction between the Delayed Trial timepoint, |lag|, and transition direction ($\hat{\beta }=-.474$, z=−2.00,p=.05). To interpret this interaction, we reverse-dummy coded the timepoint factor, setting the Delayed Trial as the reference level. Although the |lag| by transition direction interaction was significant at the Trial 5 timepoint, it was not significant at the Delayed Trial timepoint $(\hat{\beta }=.103,z=.64$, p=.52), indicating that the preference for shorter lags was similar in the forward and backward direction after a short delay.

The effect of group was not significant, indicating that participants with TBI were similarly likely to have a CRP value of 0 for a given lag transition as NC participants. There were no significant interactions between group and the Trial 1 or Delayed Trial timepoints, indicating that repetition and delay had a similar effect on the conditional response probabilities for both NC and TBI groups. There was no significant interaction between group and |lag| or between group and lag direction, indicating that these effects did not significantly differ between the two groups. All three- and four-way interactions with group, timepoint, |lag|, and transition direction were not significant, further indicating that the likelihood of having a zero CRP value did not differ by |lag| or direction for these two groups.

#### 3.3.6. Beta regression model

The second part of the model predicts the CRP value for a given lag transition for all non-zero values (i.e., when a lag transition was made, how high was its probability?) as a function of participant group, timepoint, |lag|, and direction with a random slope for |lag| by participant (Table A8).

There was a significant effect of |lag| on CRP values ($\hat{\beta }=-.481,z=-6.04,p<.001$), indicating that higher lags tended to have lower CRP values. There was a significant effect of transition direction on CRP values ($\hat{\beta }=.838,z=3.28$, p=.001), where transitions in the forward direction had higher CRP values than those in the backward direction in the NC group. Like in the zero-inflation model, a significant |lag| by direction interaction ($\hat{\beta }=-.371,z=-3.49,p<.001$) indicated that the preference for shorter lag transitions over longer ones was more pronounced in the positive than the negative direction. There was a significant negative effect of both the Trial 1 timepoint ($\hat{\beta }=-1.390,z=-6.87,p<.001$) and Delayed Trial timepoint on CRP values in the NC group ($\hat{\beta }=-.376$, z=−2.15,p=.03), indicating that NC participants tended to have lower overall conditional response probabilities at these timepoints relative to the Trial 5 timepoint. There was a significant interaction between the Trial 1 timepoint and |lag| ($\hat{\beta }=.495,z=6.44,p<.001$). The reverse-dummy coded model indicated that whereas the effect of lag was significant at the Trial 5 timepoint, it was not significant at the Trial 1 timepoint ($\hat{\beta }=.014,z=.17,p=.86$). However, there was no significant interaction between the Delayed Trial and |lag|, indicating that relative to the Trial 5 timepoint, the preference for shorter lag transitions was maintained after a short delay. The interactions between the Trial 1 and Delayed Trial timepoints with transition direction were both not significant, indicating that the tendency to have higher CRP values in the forward recall direction was similar across timepoints. Similarly, three-way interactions between the Trial 1 and Delayed Trial timepoints with |lag| and transition direction were both not significant.

There was a significant effect of group ($\hat{\beta }=-.843,z=-3.11$, p=.002), indicating that participants with TBI tended to have lower overall CRP values than NC participants. There was a significant interaction between group and the Trial 1 timepoint ($\hat{\beta }=.951,z=3.45,p=.001$). To probe this interaction, we reverse dummy coded the model, setting the TBI group as the reference level. Like in the NC group, there was a significant effect of the Trial 1 timepoint for the TBI group ($\hat{\beta }=-.439$, z=−2.340,p=.02), indicating that relative to the Trial 5 timepoint, TBI participants tended to have lower conditional response probabilities at the Trial 1 timepoint. However, the magnitude of this effect was significantly smaller in the TBI group compared to the NC group. The interaction between Group and the Delayed Trial timepoint was not significant. There was a significant interaction between Group and |lag| ($\hat{\beta }=.289,z=2.72,p=.007$). The reverse-dummy coded model indicated that although the effect of |lag| was also significant in the TBI group ($\hat{\beta }=-.192,z=-2.69,p=.007$), indicating a preference for shorter lag transitions, the magnitude of the effect was significantly reduced relative to the NC group. The interaction between group and transition direction was not significant. There was a significant three-way interaction between group, |lag|, and the Trial 1 timepoint $(\hat{\beta }=-.233$, z=−2.23,p=.03). The results of the reverse dummy coded model revealed a significant |lag| * Trial 1 interaction in the TBI group ($\hat{\beta }=.262,z=3.69,p<.001$), indicating that the preference for shorter lag transitions was more pronounced at the Trial 5 timepoint compared to the Trial 1 timepoint, but the magnitude of the effect was significantly reduced for the TBI group relative to the NC group. All other three- and four-way interactions among group, timepoint, |lag|, and transition direction were not significant.

## 4. Discussion

### 4.1. Summary

#### Recall Accuracy:

The NC group was more likely to recall narrative details than the TBI group. Relative to the No Delay timepoint, both groups were similarly likely to recall narrative details after a short delay (~20 min) but significantly less likely to recall narrative details after a long delay (~1 week). In the wordlist recall task, participants with TBI were also significantly less likely to recall a word than non-injured peers. In the NC group, the likelihood of recalling a word was significantly reduced at the Trial 1 (first presentation of wordlist) and Delayed Trial (~20 min later) timepoints relative to the Trial 5 timepoint (after five presentations of the wordlist). The difference in the likelihood of recalling a word between Trial 1 and Trial 5 timepoints was smaller in the TBI group.

#### Temporal Organization Scores:

Temporal organization scores provide insight into the overall temporal clustering of participants’ recall. Although both NC and TBI groups demonstrated above chance temporal organization in both the narrative recall and wordlist recall tasks, the temporal organization scores were significantly lower for the TBI group at the reference level timepoints (No Delay timepoint for narrative recall and Trial 5 timepoint for the wordlist recall). Thus, performance on both tasks indicates that temporal organization is present but reduced in the TBI group. The passing of time also had a significant impact on temporal organization scores. In the narrative recall task, temporal organization scores were maintained after the short 20-min delay but significantly reduced after the long one-week delay. This effect was similar for both participants with and without TBI. The effect of timepoint also followed similar patterns in the NC and TBI groups in the wordlist recall task. Temporal organization scores were significantly higher at the Trial 5 relative to Trial 1 timepoints, indicating the benefit of multiple repetitions of wordlist presentation and retrieval practice on temporal organization. This increased temporal organization was maintained after a short delay at the Delayed Trial timepoint (despite that recall accuracy was significantly lower at the Delayed Trial). Thus, while repetition of the stimulus material can improve overall temporal organization, long delays can reduce it. Finally, overall temporal organization was strongly related to recall accuracy in both tasks, indicating that participants who recalled fewer items tended to have lower temporal organization scores.

#### Lag-Conditional Response Probabilities:

Lag-conditional response probabilities provide additional insight into how participants ordered their recall in both tasks, specifically examining the temporal distances between successive recalls and whether pairs of details are recalled in a forward or backward direction. To interpret the results, we focus mostly on the results from the zero-inflation models, as these models are more representative of the entire dataset, predicting the probability that a given lag transition was made or not. For both the narrative recall and wordlist recall tasks, we replicated established temporal organization effects in the literature, indicating an asymmetrical preference for shorter lag transitions in the forward direction in the NC group. In the narrative recall task, a significant interaction between Group and |lag| in the zero-inflated model indicated that the preference to make shorter transitions was reduced in the TBI group (i.e., participants with TBI were more likely to make larger jumps between successively recalled details). In contrast, all interactions with group were not significant in the wordlist recall task, suggesting that the narrative recall task may be more sensitive to temporal (dis)organization effects in the TBI group (but note that interactions with group and |lag| were present in both tasks in the beta regression model). Participants with and without TBI were similarly affected by the passing of time (as indicated by a lack of significant interactions between group and timepoints). In the narrative recall task, participants were less likely to make shorter transitions between recalled details one week later compared to immediately after hearing the story. In the wordlist recall task, participants were more likely to make shorter transitions between recalled words after five presentations of the wordlist (Trial 5) compared to hearing it for the first time (Trial 1), providing additional evidence for a negative impact of time and a positive impact of repetition on temporal organization. The results from the beta-regression models revealed differences in the magnitude of CRP values for given transitions that occurred. These results largely paralleled those of the zero-inflation regression models, revealing a preference for short and forward lag transitions in both the narrative recall and wordlist recall tasks, but these tendencies were reduced at the Long Delay timepoint relative to No Delay timepoint in the narrative recall task and increased at the Trial 5 timepoint relative to Trial 1 timepoint in the wordlist recall task. Finally, in the wordlist recall task, participants with TBI showed both reduced practice effects (smaller effect of Trial 1 relative to Trial 5 timepoint) and a reduced preference for shorter lag transitions compared to NC participants.

*In summary*, the current study represents the first application of temporal contiguity analyses to quantify temporal organization of narrative recall in individuals with TBI. Further, examining temporal contiguity of both narrative and wordlist recall in the same study allows us to compare the pattern of findings across both experimental and naturalistic stimuli. Results across analyses and tasks converge on consistent findings: participants with TBI demonstrate reduced temporal organization of recall relative to their non-injured peers. However, like non-injured peers, they show benefits of repetition and negative impacts of delays on temporal organization. In general, relative to NC participants, participants with TBI were less likely to make short lag transitions, and conversely, more likely to make longer transitions between successively recalled items. Given that temporal organization scores were highly related to percent recall in both tasks (see Supplementary Materials B), it is likely that omissions of story details largely contributed to these larger jumps between items in the TBI group.

### 4.2. The relationship between recall performance and recall organization

The finding that participants with TBI demonstrated reduced temporal organization complements the emerging picture in the larger literature on impaired narrative discourse in TBI (Lê & Coelho, 2023; Togher et al., 2014). Participants with TBI not only recalled fewer items than their non-injured peers but also demonstrated less temporal clustering in their recall. Our finding that memory performance was strongly related to recall organization fits with previous work showing that the two are often correlated (Sederberg et al., 2010; Spillers & Unsworth, 2011). Indeed, individuals with memory impairment who recall fewer items may also make larger jumps between successively recalled items resulting in reduced temporal clustering (i.e., lower temporal organization scores) even if those items were recalled in chronological order. However, it is important to note that temporal organization scores are not dependent on the number of items recalled. For example, it is possible to only recall three story details (or words) but to recall them in the same order that they were presented, resulting in a perfect temporal organization score of 1.0. In contrast, it is also possible to recall all ten story details (or words) but to recall them in a jumbled temporal order that is no more temporally organized than chance (a temporal organization score of .5).

Nevertheless, it is difficult to disentangle the role of the declarative memory system in recall performance from its role in recall organization. On one hand, recalling fewer items leaves more opportunity for larger jumps between successively recalled items, potentially resulting in reduced temporal organization. On the other hand, failing to encode and retrieve temporal context may result in a reduced number of items recalled as these associative processes are not active during recall. It is, therefore, possible that a reduction in the number of details recalled is, at least in part, driven by a difficulty to retrieve and utilize temporal context to guide memory search during recall. Prior research has hypothesized that retrieval of the temporal context present at encoding is a useful mechanism for recalling items (Sederberg et al., 2010; Spillers & Unsworth, 2011). According to retrieved-context models (Howard & Kahana, 2002; Lohnas et al., 2015; Polyn & Cutler, 2017; Polyn et al., 2009; Sederberg et al., 2008), during encoding of a series of items (e.g., words in a list or events in a story), one’s internal contextual representation changes gradually over time. Thus, items encoded nearby in time have a more similar internal contextual representation than those with greater temporal distance in the series. During free recall, the process of memory search is driven by these internally maintained context representations. When an item is retrieved, so is the temporal context associated with the item at encoding. This retrieved context cues retrieval of neighboring items, acting as a spotlight that shifts to highlight each item and illuminating items studied nearby in time (Polyn et al., 2009).

Findings of reduced temporal organization of recall in persons with severe amnesia further highlight a strong relationship between declarative memory and recall organization. Participants with amnesia showed reduced temporal organization of their immediate recall of narratives (Evans et al., 2024) and show a diminished temporal contiguity effect (based on lag-CRP results) in a looped wordlist recall paradigm in which they were presented with the same wordlist several times in a consistent order (Palombo et al., 2019). Palombo and colleagues suggest that this impairment arises from an inability to reinstate the temporal context in which an item was encoded, such that recalled items do not serve as a reliable cue for the recall of neighboring items. They conclude that individuals with amnesia show a selective deficit in the ability to “jump back in time.” Given that individuals with moderate-severe TBI commonly have damage to the hippocampus (Palacios et al., 2013; Sharp et al., 2014; Tate & Bigler, 2000) and impairment to the declarative relational memory system (Dulas et al., 2022; Monti et al., 2013; Morrow et al., 2020; Rigon et al., 2020), it is possible that their reduced temporal organization observed in the current study is a result of a failure in recovering temporal context during recall to guide recall of subsequent items in an ordered fashion. Indeed, previous research shows that memory for temporal order is impaired in TBI, even for image sets with as few as five items (Dulas et al., 2022).

Disruption to the encoding and/or retrieval of context may underlie impairments in discourse organization in clinical populations with memory impairment. For example, individuals with schizophrenia exhibit relational memory disruption (Avery et al., 2021; Öngür et al., 2006) and reduced temporal organization of serial wordlist recall, leading the authors to propose the “context-deficit hypothesis of schizophrenia” (Polyn et al., 2015). The authors speculate that a context-specific deficit could arise from two mechanisms: One possibility, is that people with schizophrenia have damage to the mechanism that causes contextual representation to change gradually over time, resulting in a failure to create distinct contextual representations of an experience. Another possibility is that their internal contextual representation is intact, but they have damage to the mechanism that binds a studied item to that contextual representation (Polyn et al., 2015). The idea that memory deficits in schizophrenia can be characterized as a deficit in contextual processing is intriguing in its potential applications to TBI. It is possible that contextual processing deficits underlie many of the real-world communication failures that people with TBI experience that require the maintenance or integration of incoming information with context. For example, unfolding linguistic context across multiple modalities (e.g., speech and gesture), situational context, visual context, and discourse history, guide memory search and language processing. Increasingly, our group has proposed that communication challenges in TBI arise from impairments in using and processing language in context (Clough et al., 2024; Lord et al., 2024; Morrow et al., 2024).

Our ability to make strong claims about the relationship between the hippocampal declarative memory system and narrative discourse organization in the current study is limited by the diffuse nature of neural damage in TBI and lack of detailed neuropsychological and neuroanatomical data from these participants. Individuals with moderate-severe TBI present with heterogenous patterns of diffuse neural injury and cognitive impairments (Covington & Duff, 2021). Given the high interconnectivity between the frontal lobe and medical temporal lobe (Barnett et al., 2021; Thierry et al., 2000) paired with the diffuse nature of neuronal injury and damage to white matter tracts in TBI (Hayes et al., 2016), it is likely that disruptions to multiple cognitive functions contribute to discourse disorganization. However, the current study, along with a growing body of work in cognitive neuroscience (Baldassano et al., 2017; Cohn-Sheehy, 2023; Cohn-Sheehy et al., 2021; Evans et al., 2024; Kurczek & Duff, 2011), suggests that hippocampal declarative memory is not only one of these contributors but, in fact, plays a critical role in narrative recall ability and organization.

### 4.3. Effects of repetition and time

Temporal organization was present but reduced in the TBI group. One important clinical application is understanding whether temporal contiguity effects are malleable. Examining the effects of timepoint in both the wordlist recall and narrative recall task sheds insights into this question. In the narrative recall task, we examined temporal organization across three timepoints: immediately after hearing the story, after a short 20-min delay, and after a long one-week delay. The results showed that the long delay negatively impacted temporal organization to a similar magnitude for both NC and TBI groups. This is in line with prior research which has shown that temporal contiguity is often present but may be reduced at longer delays (see Healey et al., 2019 for review). This would be expected if over time the internal contextual representation of items becomes less distinct than immediately after encoding, making it a less reliable cue for guiding recall. Thus, passing time has a negative impact on temporal organization of free recall.

The wordlist recall analysis also included three timepoints: Trial 1 represents participants’ recall after hearing the wordlist for the first time, Trial 5 represents participants’ recall after five repeated presentations of the wordlist in the same order, and the Delayed Trial represents participants’ recall after a short 20-min delay. The contrast between Trial 1 and Trial 5 showed that temporal organization increases with repeated presentations and retrieval practice of the wordlist, indicating that repetition is a strategy that can be used to improve temporal organization. This effect was of a similar magnitude in the NC and TBI groups. Indeed, repetition is an example of an internal memory strategy that can be leveraged to compensate for memory impairments in TBI (Velikonja et al., 2014, 2023). In contrast, when the presentation order is varied over repeated word list presentations, this weakens representation of the temporal context and can lead to the interruption of typical temporal contiguity effects (Hong et al., 2024). Within the retrieved-context model, each presentation of the wordlist in the same order may reinforce the contextual representations across items as neighboring items can become more strongly associated. If presentation of the wordlist order had varied it would have likely disrupted these contextual representations and created confounding associations. In the present work, after a short delay of 20 min, temporal organization was preserved for the wordlist recall (similar to the finding that temporal organization was maintained at the short delay in the narrative recall task). Although temporal organization was maintained at short delays, it is unclear whether repetition would mitigate the negative effect of a long delay on temporal organization. It is an open question how repetition along with other memory strategies such as spaced retrieval (Velikonja et al., 2014, 2023) throughout the period of the long delay may be leveraged to reduce the negative impact of time on temporal organization, particularly for supporting recall organization for populations with memory impairment.

### 4.4. Toward more ecologically valid measures of recall organization

Temporal contiguity analyses are staples of the memory literature which have traditionally used wordlist recall paradigms (Sederberg et al., 2010; Healey et al., 2019; Howard & Kahana, 2002; Polyn et al., 2009). These types of free recall tasks show robust and replicable memory effects but have low ecological validity as we seldom need to recall unrelated wordlists in the real-world. The current study is limited in its ability to directly compare temporal organization in the narrative and wordlist recall tasks due to differences in grain size of recall items (i.e., details in narrative recall task versus words in wordlist recall task) and single versus repeated exposure of studied items in the narrative and wordlist recall tasks, respectively. Nevertheless, the temporal organization scores (see Fig. 3) and +1 lag-CRP values (see Fig. 4) tended to be higher in the narrative recall task than the wordlist recall task. Extending this paradigm to narrative recall may better reflect participants’ functional memory abilities. That said, there are important differences between wordlist recall and narrative recall tasks that may limit the ability of the temporal contiguity analyses used in the current study to meaningfully capture all aspects of narrative recall organization. Wordlist recall tasks often employ lists of unrelated words so that participants rely only on encoded temporal context, rather than meaningful relationships among the words, to guide the process of recall. When lists with inherent semantic categories or relations have been employed, these paradigms either compare recall of lists that are all from one category to uncategorized lists or use mixed category lists that intentionally space same category items to either compliment or contrast temporal organization (Hong et al., 2024; Polyn et al., 2009, 2011; Ward & Tan, 2023). In contrast, narratives typically have both an underlying temporal and semantic structure where story details are clustered into a series of larger episodes consisting of logical relationships between events and characters. Thus, the temporal and semantic contexts in a narrative that is told chronologically (such as the stories in our study) do not create differing or competing predictions about recall order. Therefore, when the present participants recalled the narrator’s stories, they could use both temporal and semantic context to organize narrative structure and facilitate recall.

Although memory encoding is associated with a gradually changing temporal context (Howard & Kahana, 2002; Kahana et al., 2008; Lohnas et al., 2015; Polyn et al., 2009), continuous experiences are organized into discrete episodes that shape the organization of episodic memory (Clewett & Davachi, 2017; DuBrow & Davachi, 2013; Heusser et al., 2018; Nolden et al., 2024; Zacks et al., 2007; Zheng et al., 2022). Episodic boundaries (also referred to as event boundaries) mark transitions between one episode and another. For example, preparing a Halloween costume and attending a Halloween party might be perceived as two distinct episodes or events within a story, each containing their own series of details. Given that narratives have a more complex hierarchical structure of details organized into episodes that in turn compose the narrative arc, characterizing the temporal organization of narrative recall may benefit from an approach that quantifies the degree to which temporal organization is present within and across episodic boundaries. This kind of hierarchical temporal contiguity analysis would build on current measures of story grammar in TBI which calculate the proportion of utterances in a narrative organized into episodes (Coelho et al., 2012; Coelho, 2002; Hay & Moran, 2005; Peach & Hanna, 2021).

There are increasing examples of studies examining temporal organization taking these episodic boundaries into consideration. For example, Pu et al. (2022) presented participants with sequentially presented pictures in which episodic boundaries were marked by changes to the frame color of the pictures. They showed that temporal organization was greater for items within an episode compared to across episodes, suggesting that temporal context associated with encoded items resets at episodic boundaries (Pu et al., 2022). We would, therefore, expect temporal organization of story details to be greater within than across episodes. Other studies have shown that temporal continguity analyses can by applied within episodic boundaries marked by changes in attentional states during encoding of pictures of common objects (Jayakumar et al., 2023), and Evans (2025) proposed a hierarchical model of temporal organization of conversation recall in which information units are clustered within conversational topics. A hierarchical approach to temporal organization of narrative recall would be highly useful for capturing plot-relevant temporal organization effects where semantic content defines contextual boundaries. The current study is not positioned to address this question due to the relatively small number of details per narrative (10 or 12) and a lack of clear event boundaries distinct from these details, restricting the number of story details clustered within a given episode. Future studies should consider designing narrative stimuli with this in mind to better control the number of story details clustered within each episode to better capture these complex aspects of narrative structure. For example, Ezzyat and Davachi (2011) examined associative memory for imformation within and across episodic boundaries in narratives composed of thirty-eight sentence each using transitions like, “A while later…” to indicate temporal event boundaries between episodes. The degree to which narrative recall in TBI is organized by temporal and semantic clustering thus remains an exciting an open question.

Finally, in the current study, participants passively listened to pre-recorded stories from a narrator in a video before being prompted to recall them. This is analogous to hearing a story from a friend. However, it is an open question how the temporal organization of narrative recall might differ if participants with TBI were asked to generate their own story based on a sequence of events they experienced. In such a study, one could compare the order in which participants experienced events to the order in which they retold them to better understand the relationships between event memory, narrative discourse, and temporal organization. Increasing the ecological validity of lab-based experiments is critical to better reflect and model how discourse interacts with contextual factors and cognitive demands that reflect everyday life.

### 4.5. Conclusion

We introduced a novel technique for characterizing the temporal organization of narrative organization in individuals with moderate-severe TBI using temporal contiguity analyses traditionally used for serial wordlist recall. Participants with moderate-severe TBI showed above chance but significantly reduced temporal organization of narrative recall and wordlist recall relative to non-injured peers, indicated by reduced overall temporal organization scores and reduced tendency of making shorter lag transitions between pairs of successively recalled details. A long one-week delay had a negative impact on temporal organization whereas repetition and retrieval practice improved temporal organization. Overall temporal organization scores were strongly linked to recall performance, suggesting that the declarative relational memory system plays an important role in not only the total number of items recalled, but also in the ability to use temporal context to guide memory search during free recall to retrieve items in order. The current findings highlight that disruption to declarative memory may be a strong contributing factor underlying impaired narrative discourse organization in individuals with moderate-severe TBI.

## Supplementary Material

1

2

Supplementary data to this article can be found online at https://doi.org/10.1016/j.cortex.2025.06.007.

## Figures and Tables

**Table 1 –:** Demographic information for NC and TBI participants.

	NC	TBI	*p-value*
Sample size (*n*)	60	60	
Age at study [mean (*SD*)]	39.95 (10.39)	40.13 (10.60)	.93
Sex = Male [*n* (%)]	30 (50.00%)	30 (50.00%)	1.00
Education [mean (SD)]	14.97 (2.43)	14.93 (2.51)	.93
Time post-injury [mean (*SD*)]	NA	74.62 (74.23)	NA
Injury etiology [*n* (%)]			NA
Assault	NA	3 (5.00%)	
Fall from height	NA	5 (8.33%)	
Ground-level fall	NA	4 (6.67%)	
Motor vehicle accident	NA	27 (45.00%)	
Motorcycle and snowmobile collisions	NA	8 (13.33%)	
Non-motorized vehicle accident	NA	5 (8.33%)	
Other	NA	3 (5.00%)	
Pedestrian struck by vehicle	NA	3 (5.00%)	
Struck by object	NA	2 (3.33%)	

*Note.* Age = years. Education indicates years of highest degree earned. Time post-injury presented in months. NA = not applicable.

**Table 2 –:** Mean narrative temporal organization scores of narrative recall for each group and timepoint.

Group	Mean (SD)	Compared to chance (.5)

NC	.857 (.13)	*t*(719) = 76.37, *p* < .001
TBI	.794 (.17)	*t*(696) = 45.93, *p* < .001

Timepoint	Mean (*SD*)	Compared to chance (.5)

No delay	.850 (.13)	*t*(478) = 60.30, *p* < .001
Short delay	.840 (.14)	*t*(476) = 52.53, *p* < .001
Long delay	.787 (.18)	*t*(460) = 34.92, *p* < .001

**Table 3 –:** Mean temporal organization scores of word list recall for each group and timepoint.

Group	Mean (*SD*)	Compared to chance (.5)

NC	.734 (.17)	*t*(148) = 17.36, *p* < .001
TBI	.680 (.16)	*t*(170) = 14.27, *p* < .001

Timepoint	Mean (*SD*)	Compared to chance (.5)

Trial 1	.645 (.15)	*t*(106) = 9.97, *p* < .001
Trial 5	.749 (.16)	*t*(106) = 15.80, *p* < .001
Delayed	.722 (.17)	*t*(106) = 13.46, *p* < .001

## Appendix

**Table A1 –:** Probability of recalling a story detail as a function of group and timepoint; Results from binomial mixed effect model fit by maximum likelihood

Fixed Effects	Estimate	SE	*z*-value	*p*-value
(Intercept)	1.538	.161	9.542	<.001
Group	−.596	.153	−3.892	<.001
Short delay	.005	.073	.073	.942
Long delay	−.433	.070	−6.227	<.001
Group * short delay	−.174	.097	−1.785	.074
Group * long delay	−.180	.095	−1.902	.057
Random Effects	Variance		SD	

Participant (intercept)	.556		.746	
Story (intercept)	.056		.236	

*Note.* NC group and No Delay timepoint are dummy coded as the reference level.

**Table A2 –:** Probability of recalling a word as a function of group and timepoint; Results from binomial mixed effect model fit by maximum likelihood

Fixed Effects	Estimate	SE	*z*-value	*p*-value
(Intercept)	2.816	.252	11.174	<.001
Group	−1.023	.238	−4.299	<.001
Short delay	−2.421	.163	−14.897	<.001
Long delay	−.595	.176	−3.381	.001
Group * short delay	.474	.203	2.342	.019
Group * long delay	.083	.217	.381	.704
Random Effects	Variance		SD	

Participant (intercept)	.742		.861	
Word (intercept)	.426		.652	

*Note.* NC group and No Delay timepoint are dummy coded as the reference level.

**Table A3 –:** Narrative temporal organization scores as a function of group and timepoint; Results from linear mixed model fit by REML

Fixed Effects	Estimate	SE	*t*-value	*p*-value
(Intercept)	.883	.020	43.114	<.001
Group	−.066	.018	−3.604	<.001
Short delay	−.009	.011	−.834	.405
Long delay	−.067	.011	−6.085	<.001
Group * short delay	−.005	.016	−.299	.765
Group * long delay	−.005	.016	−.331	.741
Random Effects	Variance		SD	

Participant (intercept)	.006		.080	
Story (intercept)	.001		.031	

*Note.* NC group and No Delay timepoint are dummy coded as the reference level.

**Table A4 –:** Wordlist Temporal organization scores as a function of group and timepoint; Results from linear regression model

Fixed Effects	Estimate	SE	*t*-value	*p*-value
(Intercept)	.783	.023	34.717	<.001
Group	−.064	.031	−2.075	.039
Trial 1	−.128	.032	−4.024	<.001
Delayed trial	−.019	.032	−.585	.559
Group * trial 1	.046	.044	1.057	.291
Group * delayed trial	−.017	.044	−.383	.702

*Note.* NC group and Trial 5 timepoint are dummy coded as the reference level.

**Table A5 –:** Conditional-Response Probability as a function of group, timepoint, lag transition, and direction for narrative recall task; Results from zero-inflation model

Fixed Effects	Estimate	SE	z-value	*p*-value
(Intercept)	−2.392	.286	−8.351	<.001
Group	.856	.276	3.102	.002
Short delay	.430	.235	1.830	.067
Long delay	.965	.221	4.376	<.001
Lag	1.342	.128	10.470	<.001
Direction	−4.606	.343	−13.415	<.001
Group * short delay	−.319	.318	−1.003	.316
Group * long delay	−.310	.301	−1.029	.303
Group * lag	−.320	.105	−3.045	.002
Short delay * lag	−.168	.095	−1.769	.077
Long delay * lag	−.466	.087	−5.375	<.001
Group * direction	.648	.455	1.424	.154
Short delay * direction	−.143	.470	−.304	.761
Long delay * direction	.522	.441	1.184	.236
Lag * direction	.804	.141	5.703	<.001
Group * short delay * lag	.145	.127	1.139	.255
Group * long delay * lag	.180	.117	1.536	.125
Group * short delay * direction	.046	.635	.073	.942
Group * long delay * direction	.154	.601	.256	.798
Group * lag * direction	−.235	.184	−1.277	.202
Short delay * lag * direction	.146	.189	.769	.442
Long delay * lag * direction	.001	.174	.006	.995
Group * short delay * lag * direction	−.154	.255	−.607	.544
Group * long delay * lag * direction	−.107	.234	−.457	.648
Random Effects	Variance		SD	

Participant (intercept)	.739		.860	
Lag slope by participant	.076		.276	
Story (intercept)	.161		.401	
Lag slope by story	.040		.201	

*Note.* NC group and No Delay timepoints are dummy coded as the reference level. Lag represents the absolute lag transition value (|1|, |2|, |3|, |4|, |5|). Direction is effects coded (negative = −.5, positive = .5).

**Table A6 –:** Conditional-Response Probability as a function of group, timepoint, lag transition, and direction for narrative recall task; Results from beta regression model

Fixed Effects	Estimate	SE	z-value	*p*-value
(Intercept)	.811	.230	3.518	<.001
Group	−.609	.237	−2.571	.010
Short delay	.002	.184	.012	.990
Long delay	−.436	.183	−2.383	.017
Lag	−.161	.105	−1.525	.127
Direction	1.853	.323	5.728	<.001
Group * short delay	.103	.262	.392	.695
Group * long delay	.015	.257	.059	.953
Group * lag	.204	.119	1.712	.087
Short delay * lag	.021	.101	.211	.833
Long delay * lag	.173	.097	1.791	.073
Group * direction	−.490	.446	−1.099	.272
Short delay * direction	.084	.367	.229	.819
Long delay * direction	−.840	.366	−2.293	.022
Lag * direction	−1.703	.191	−8.897	<.001
Group * short delay * lag	−.069	.139	−.499	.618
Group * long delay * lag	.049	.132	.375	.708
Group * short delay * direction	−.507	.523	−.969	.332
Group * long delay * direction	.453	.513	.883	.377
Group * lag * direction	.559	.258	2.165	.030
Short delay * lag * direction	.015	.201	.076	.939
Long delay * lag * direction	.613	.194	3.164	.002
Group * short delay * lag * direction	.245	.278	.882	.378
Group * long delay * lag * direction	−.416	.264	−1.577	.115
Random Effects	Variance		SD	

Participant (intercept)	.617		.785	
Lag slope by participant	.099		.314	
Direction slope by participant	1.703		1.305	
Lag * direction slope by participant	.696		.834	
Story (intercept)	.095		.308	
Lag slope by story	.040		.201	

*Note.* NC group and No Delay timepoints are dummy coded as the reference level. Lag represents the absolute lag transition value (|1|, |2|, |3|, |4|, |5|). Direction is effects coded (negative = −.5, positive = .5).

**Table A7 –:** Conditional-Response Probability as a function of group, timepoint, lag transition, and direction for wordlist recall task; Results from zero-inflation model

Fixed Effects	Estimate	SE	z-value	p-value
(Intercept)	−2.098	.275	−7.628	.000
Group	−.047	.371	−.127	.899
Trial 1	1.124	.361	3.111	.002
Delayed trial	.160	.373	.430	.667
Lag	.789	.087	9.032	<.001
Direction	−1.839	.542	−3.394	.001
Group * trial 1	.454	.490	.927	.354
Group * delayed trial	.698	.497	1.403	.160
Group * lag	−.049	.116	−.424	.671
Trial 1 * lag	−.282	.116	−2.437	.015
Delayed trial * lag	−.090	.119	−.754	.451
Group * direction	.283	.731	.388	.698
Trial 1 * direction	−.449	.722	−.622	.534
Delayed trial * direction	1.001	.746	1.342	.180
Lag * direction	.577	.174	3.312	.001
Group * trial 1 * lag	.026	.157	.166	.868
Group * delayed trial * lag	−.145	.157	−.926	.354
Group * trial 1 * direction	.223	.980	.228	.820
Group * delayed trial * direction	−.448	.994	−.451	.652
Group * lag * direction	−.102	.232	−.440	.660
Trial 1 * lag * direction	−.026	.231	−.112	.911
Delayed trial * lag * direction	−.474	.237	−1.997	.046
Group * trial 1 * lag * direction	−.050	.315	−.159	.874
Group * delayed trial * lag * direction	.217	.314	.692	.489
Random Effects	Variance		SD	

Participant (intercept)	.108		.328	

*Note.* NC group and Trial 5 timepoint are dummy coded as the reference level. Lag represents the absolute lag transition value (|1|, |2|, |3|, |4|, |5|). Direction is effects coded (negative = −.5, positive = .5).

**Table A8 –:** Conditional-Response Probability as a function of group, timepoint, lag transition, and direction for wordlist recall task; Results from beta regression model

Fixed Effects	Estimate	SE	z-value	*p*-value
(Intercept)	.714	.198	3.610	.000
Group	−.843	.271	−3.112	.002
Trial 1	−1.390	.202	−6.871	<.001
Delayed trial	−.376	.175	−2.153	.031
Lag	−.481	.080	−6.042	<.001
Direction	.838	.255	3.281	.001
Group * trial 1	.951	.276	3.449	.001
Group * delayed trial	.372	.241	1.545	.122
Group * lag	.289	.106	2.719	.007
Trial 1 * lag	.495	.077	6.438	<.001
Delayed trial * lag	.092	.071	1.293	.196
Group * direction	−.078	.349	−.223	.824
Trial 1 * direction	−.372	.401	−.928	.353
Delayed trial * direction	−.327	.352	−.930	.352
Lag * direction	−.371	.106	−3.492	<.001
Group * trial 1 * lag	−.233	.105	−2.231	.026
Group * delayed trial * lag	−.091	.096	−.956	.339
Group * trial 1 * direction	−.175	.553	−.317	.751
Group * delayed trial * direction	−.100	.485	−.205	.837
Group * lag * direction	−.064	.143	−.447	.655
Trial 1 * lag * direction	.032	.153	.211	.833
Delayed trial * lag * direction	.022	.144	.156	.876
Group * trial 1 * lag * direction	.057	.211	.270	.787
Group * delayed trial * lag * direction	.084	.194	.434	.665
Random Effects	Variance		SD	

Participant (intercept)	1.136		1.066	
Lag Slope by participant	.162		.403	

*Note.* NC group and Trial 5 timepoint are dummy coded as the reference level. Lag represents the absolute lag transition value (|1|, |2|, |3|, |4|, |5|). Direction is effects coded (negative = −.5, positive = .5).
