# Supplementary material for: Reduced temporal organization of narrative recall in adults with moderate-severe traumatic brain injury
Source: Cortex. Author manuscript; Available in PMC 2026 May 30. (PMC13222038; doi:10.1016/j.cortex.2025.06.007)
Supplement: 1 [file NIHMS2168990-supplement-1.docx]

**Supplementary Materials B: Relationship between recall accuracy and temporal organization scores**

Narrative Recall Task

For the narrative recall task, participants with TBI had lower temporal organization scores than NC peers, and both groups had reduced temporal organization scores at the Long- compared to No-Delay timepoint (Analysis 3.2.2). However, these results parallel the findings of reduced recall accuracy in the TBI group and Long Delay timepoint (Analysis 3.1.1). To better understand whether reduced temporal organization scores in the TBI group and the Long Delay timepoint were related to poorer recall performance in the narrative recall task, we re-ran the model, adding Percent Recall for each story (grand mean-centered) as a covariate (Table S1). Indeed, there was a significant effect of Percent Recall ($\hat{\beta}$ = 0.546, *t* = 32.38, *p* < .001), where participants who recalled a greater percentage of the story details tended to have higher temporal organization scores. When accounting for differences in narrative recall percentage, the effect of participant group was no longer significant, suggesting that the negative impact of TBI on temporal organization is not independent of recall accuracy within the task. However, the effect of the Long Delay remained significant ($\hat{\beta}$ = -0.027, *t* = -2.98, *p* = .003), suggesting that a one-week delay had a negative impact on temporal organization above and beyond its impact on reduced recall performance. The effect of the Short Delay and interactions between group and timepoints were not significant.

| **Table S1.** *Narrative temporal organization scores as a function of group and timepoint, including percent recall as a covariate; Results from Linear mixed model fit by REML* | | | | |
| --- | --- | --- | --- | --- |
| *Fixed Effects* | *Estimate* | *SE* | *t-value* | *p-value* |
| (Intercept) | 0.838 | 0.017 | 50.354 | <.001 |
| Group | -0.010 | 0.011 | -0.914 | 0.362 |
| Short Delay | -0.009 | 0.009 | -1.036 | 0.300 |
| Long Delay | -0.027 | 0.009 | -2.983 | 0.003 |
| Percent Recall | 0.546 | 0.017 | 32.378 | <.001 |
| Group * Short Delay | 0.012 | 0.013 | 0.959 | 0.338 |
| Group * Long Delay | 0.013 | 0.013 | 1.009 | 0.313 |
| *Random Effects* | *Variance* | | *SD* | |
| Participant (intercept) | 0.001 | | 0.029 | |
| Story (intercept) | 0.001 | | 0.030 | |
| *Note.* NC group and No Delay timepoint are dummy coded as the reference level. Percent Recall is mean-centered. | | | | |

Wordlist Recall Task

For the wordlist recall task, participants with TBI had lower temporal organization scores than NC peers, and both groups had reduced temporal organization scores at the Trial 1 compared to Trial 5 timepoint (Analysis 3.2.4). However, these results parallel the findings of reduced recall accuracy in the TBI group and Trial 1 timepoint (Analysis 3.1.2). To better understand whether reduced temporal organization scores in the TBI group and the Trial 1 timepoint were related to poorer recall performance, we re-ran the model, adding Percent Recall for each wordlist recall attempt (grand mean-centered) as a covariate (Table S2). Indeed, there was a significant effect of percent recall ($\hat{\beta}$ = 0.426, *t* = 8.57, *p* < .001), where participants who recalled a greater percentage of the wordlist tended to have higher temporal organization scores. When accounting for differences in wordlist recall, the effect of participant group and Trial 1 timepoint were no longer significant, suggesting that these effects were not independent from memory performance in the task. The effect of the Delayed timepoint and interactions between participant group and timepoints were not significant.

| **Table S2.** *Wordlist temporal organization scores as a function of group and timepoint, including percent recall as a covariate; Results from linear regression model* | | | | |
| --- | --- | --- | --- | --- |
| *Fixed Effects* | *Estimate* | *SE* | *t-value* | *p-value* |
| (Intercept) | 0.703 | 0.022 | 31.371 | <.001 |
| Group | -0.022 | 0.028 | -0.770 | 0.442 |
| Trial 1 | 0.016 | 0.033 | 0.474 | 0.636 |
| Delayed Trial | 0.003 | 0.029 | 0.120 | 0.904 |
| Percent Recall | 0.426 | 0.050 | 8.565 | <.001 |
| Group * Trial 1 | 0.050 | 0.039 | 1.266 | 0.206 |
| Group * Delayed Trial | -0.007 | 0.039 | -0.178 | 0.859 |
| *Note.* NC group and Trial 5 timepoint are dummy coded as the reference level. Percent Recall is mean-centered. | | | | |
